# INCREASING RISK FOR ELDER MISTREATMENT? A QUALITATIVE STUDY OF CAREGIVER EXPERIENCES DURING COVID-19

**DOI:** 10.1093/geroni/igac059.663

**Published:** 2022-12-20

**Authors:** Kelly Marnfeldt, Lilly H Estenson, Julia Marinez, Zach Gassoumis, Kathleen Wilber

**Affiliations:** University of Southern California, Santa Monica, California, United States; University of Southern California, Pasadena, California, United States; University of Southern California, Los Angeles, California, United States; Keck School of Medicine, USC, Los Angeles, California, United States; University of Southern California, Los Angeles, California, United States

## Abstract

Family caregivers (CGs) of community-dwelling older adults faced unprecedented psychosocial challenges in the face of the COVID-19 pandemic. Social isolation, lack of social support, truncated care networks, financial hardship and other factors put many older adults at risk for elder mistreatment (EM). Even in normal times, incidents of EM are only identified and formally reported in about one out of every 24 cases. Social distancing restrictions in the caregiving context, including decreased access to healthcare providers, rendered older adults with higher dependency needs inordinately vulnerable to abuse and neglect. We collected qualitative data on the experiences of 24 CGs receiving telephonic support sessions between March 2020 and February 2021. Data from 100 distinct post-session case notes were analyzed using thematic content analysis to generate codes and themes. Caregivers reported rapid deterioration of care receiver’s (CRs) physical health, resulting in higher levels of dependency. Declines in mental health and cognitive impairment, including behavioral issues, were an indicator of stress for both CR and CG. CGs reported lack of access to services and social supports, fractured care networks, and increased feelings of isolation. Preliminary content analysis indicates that serious risk factors for EM were present across all 13 months of the study, and those risk factors continued or worsened over time. Findings suggest that the unintended consequences of the public health response to COVID-19 may have amplified the risk for EM in vulnerable community-dwelling older adults.

